# Structural Basis of Cooperativity in Human UDP-Glucose Dehydrogenase

**DOI:** 10.1371/journal.pone.0025226

**Published:** 2011-10-03

**Authors:** Venkatachalam Rajakannan, Hui-Sun Lee, Seon-Ha Chong, Han-Bong Ryu, Ji-Young Bae, Eun-Young Whang, Jae-Wan Huh, Sung-Woo Cho, Lin-Woo Kang, Han Choe, Robert C. Robinson

**Affiliations:** 1 Institute of Molecular and Cell Biology, Agency for Science, Technology and Research, Singapore, Singapore; 2 Department of Physiology, University of Ulsan College of Medicine, Seoul, Korea; 3 Bio-Medical Institute of Technology, University of Ulsan College of Medicine, Seoul, Korea; 4 Department of Biochemistry and Molecular Biology, University of Ulsan College of Medicine, Seoul, Korea; 5 Department of Advanced Technology Fusion, Kunkuk University, Seoul, Korea; Russian Academy of Sciences-, Institute for Biological Instrumentation, Russian Federation

## Abstract

**Background:**

UDP-glucose dehydrogenase (UGDH) is the sole enzyme that catalyzes the conversion of UDP-glucose to UDP-glucuronic acid. The product is used in xenobiotic glucuronidation in hepatocytes and in the production of proteoglycans that are involved in promoting normal cellular growth and migration. Overproduction of proteoglycans has been implicated in the progression of certain epithelial cancers, while inhibition of UGDH diminished tumor angiogenesis *in vivo*. A better understanding of the conformational changes occurring during the UGDH reaction cycle will pave the way for inhibitor design and potential cancer therapeutics.

**Methodology:**

Previously, the substrate-bound of UGDH was determined to be a symmetrical hexamer and this regular symmetry is disrupted on binding the inhibitor, UDP-α-D-xylose. Here, we have solved an alternate crystal structure of human UGDH (hUGDH) in complex with UDP-glucose at 2.8 Å resolution. Surprisingly, the quaternary structure of this substrate-bound protein complex consists of the open homohexamer that was previously observed for inhibitor-bound hUGDH, indicating that this conformation is relevant for deciphering elements of the normal reaction cycle.

**Conclusion:**

In all subunits of the present open structure, Thr131 has translocated into the active site occupying the volume vacated by the absent active water and partially disordered NAD^+^ molecule. This conformation suggests a mechanism by which the enzyme may exchange NADH for NAD^+^ and repolarize the catalytic water bound to Asp280 while protecting the reaction intermediates. The structure also indicates how the subunits may communicate with each other through two reaction state sensors in this highly cooperative enzyme.

## Introduction

UDP-glucose 6-dehydrogenase (UGDH; EC 1.1.1.22) is the sole human enzyme that converts UDP-α-D-glucose (UDP-glucose) to UDP-α-D-glucuronic acid (UDP-glucuronic acid), an intermediate sugar in carbohydrate metabolism [1]. UDP-glucuronic acid is incorporated into hyaluronan, chondroitin sulfate, heparan sulfate, and glycosaminoglycans. C5 epimerization leads to iduronate for inclusion in heparan sulfate and dermatan sulfate. Such extracellular matrix carbohydrates promote normal cellular growth, embryogenesis and adult organism physiology [2], [3], [4]. In addition, UDP-glucuronic acid is used in the glucuronidation of many molecules including drugs, nuclear hormones, retinoids, bile acids, bilirubin, and fatty acid derivatives by UDP-glucuronosyl transferase in hepatocytes [5]. UGDH is also implicated in tumor progression and osteoarthritis [6], [7], [8]. Overproduction of proteoglycans has been observed in the progression of epithelial cancers such as colon, breast, and prostate [9], [10], [11]. Inhibition of hUGDH has been demonstrated to diminish tumor angiogenesis in vivo [12], while UGDH gene disruption in zebrafish led to a heart valve defect [13].

**Figure 1:**
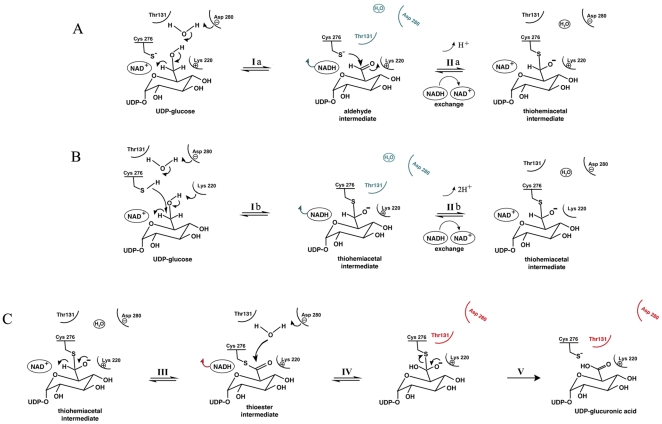
Mechanism of hUGDH catalysis. (A) In the first phase of the mechanism established from the structure of bacterial UGDH (Easley et al., 2007), a water molecule, activated by Asp280, is predicted to be the general acid/base catalyst that abstracts a proton from the C6′ hydroxyl to initiate oxidation. Mutation of Asp280 to Asn is detrimental to catalysis. An aldehyde intermediate is transiently formed at the active site (Ia). This aldehyde has not been detected experimentally [14], however, added aldehyde functions as a substrate for the second oxidation [45]. The intermediate is then proposed to rapidly convert to the thiohemiacetal adduct (IIa), via Cys276, without being released and the second NAD^+^ to replace the first reduced cofactor, NADH. (B) In an updated mechanism (Ib and IIb), the reaction proceeds directly to the thiohemiacetal adduct without proceeding through the aldehyde intermediate [28]. (C) In the second phase of both mechanisms, the thiohemiacetal is then oxidized to the thioester via transfer of hydrogen to NAD^+^ (III). Finally the acid product is released through spontaneous hydrolysis [46], [47]. Here the water bound to Asp280 is highlighted as the probable active water molecule. Evidence for a covalent thiohemiacetal (II) lies in the observation that the second deprotonation step (III) is reversible while the overall conversion to acid is irreversible (V) [38]. Mutation of Cys276 to serine led to the build up of covalently attached adduct, however, the C276A mutation, while not able to proceed to completion from UDP-glucose, was able to catalyze the oxidation of the aldehyde intermediate [45]. Lys220 provides charge stabilization to the anionic transition state during the second oxidation step (III), and for the course of the hydrolysis of the thioester (IV and V). Mutation of Lys220 significantly, but not completely, reduced the enzyme function suggesting that it does not form a Schiff's base [34], [48]. Features in blue and red indicate that Thr131 and Asp280 coordinate the movements of NAD^+^/NADH and the active water molecule.

Bovine UGDH was first identified in the liver [14], later purified [15] and the amino acid sequence determined [16]. Molecular cloning of hUGDH revealed it to be a 494-amino acid protein that shares 98% identity to bovine UGDH [17], [18], [19]. hUGDH is a hexamer of 57-kDa subunits that assembles into a trimer of dimers. UGDH is a member of a small group of NAD^+^-dependent four-electron-transfer dehydrogenases. Substrate, inhibitor and product binding experiments and chemical modification studies all point towards three subunits being catalytically active at any one time, mirroring the structural assembly [1], [20], [21], [22], [23]. UGDH is also found in prokaryotes, producing the UDP-glucuronic acid that is essential for the synthesis of antiphagocytic capsular polysaccharides [24], [25], [26]. The crystal structures of dimeric S. pyogenes UGDH provided the structural basis for the reaction cycle (Fig. 1A,C) [27], which was further refined through elucidation of hUGDH hexameric structures (Fig. 1B,C) [28]. UGDH catalyzes two NAD^+^-dependent oxidations of UDP-glucose, the first reversible, to yield UDP-glucuronic acid in an overall irreversible process [29], [30]. The structures of hUGDH in complex with NAD^+^ and UDP-glucose, a E161Q hUGDH mutant bound to the thiohemiacetal intermediate, hUGDH in complex with the product UDP-glucuronate and a T131A hUGDH mutant in the apo form (PDB codes 2Q3E, 3KHU, 2QG4 and 3ITK, respectively) are all closed hexamers [28]. In contrast, inhibitor-bound hUGDH adopts an open hexameric conformation in which the three-fold symmetry has been broken [31]. Here, we have solved an alternate substrate-bound structure of hUGDH, which reveals the asymmetrical open hexameric quaternary conformation that was previously observed in the inhibitor-bound structure. Conformational differences between open and closed hexamer conformations suggest structural-based mechanisms for the repolarization of the active water molecule, reaction intermediate protection, and cooperativity between subunits.

## Results

### Structure of hUGDH

**Figure 2:**
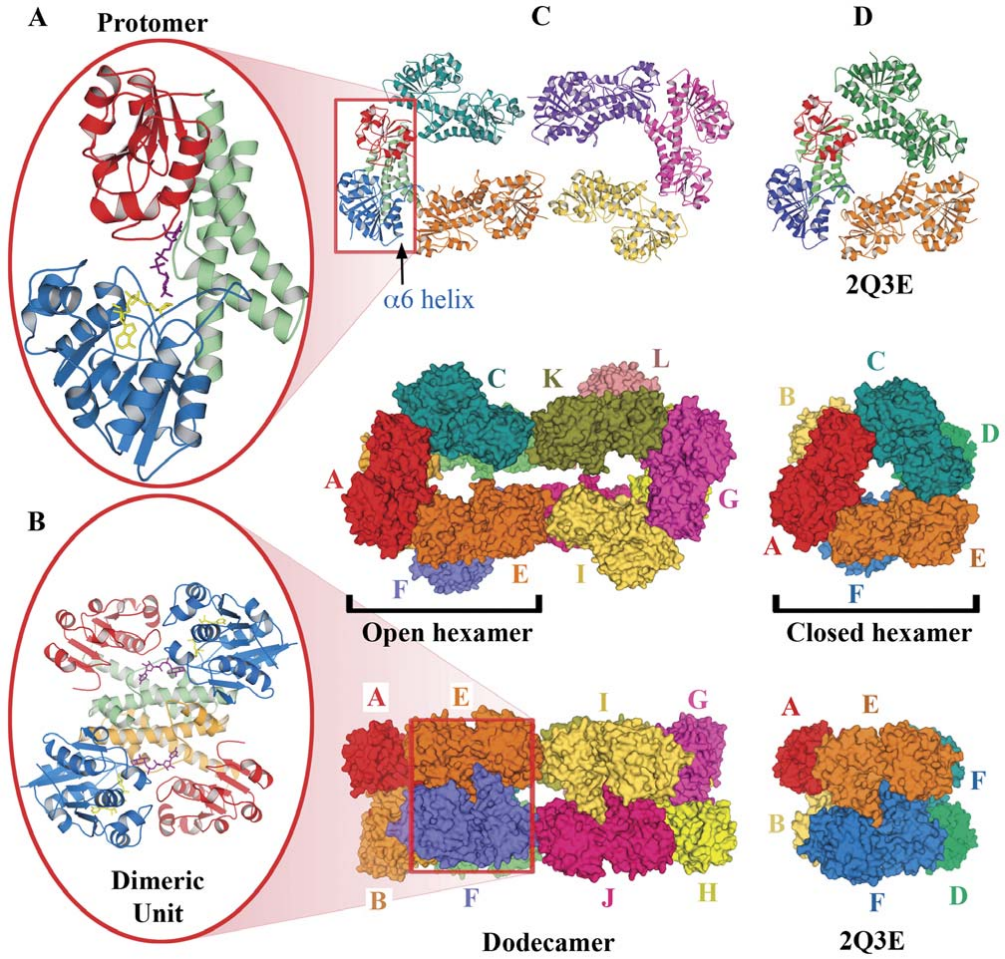
Structure of hUGDH. (A) Schematic representation of a hUGDH protomer taken from the dodecamer. N-terminal, central and C-terminal domains are colored blue, green and red, respectively. UDP glucose is shown in purple, NAD^+^ in yellow. (B) The closely associated dimeric unit taken from the dodecamer. The second copy of the central domain is tinted orange. (C) The dodecameric hUGDH arrangement that is present in the crystal asymmetric unit comprised of two open hexamers. Upper panel shows the top layer of the dodecamer. Central and lower panels depict the full dodecamer in space filled representation. The twisted conformation is evident from the lower panel. (D) For comparison, a typical symmetric hexamer of hUGDH taken from PDB code 2Q3E. Movies S1 and S2 show morphs between these two structures. Protein representations were generated here, and in the figures that follow, using PYMOL (http://pymol.sourceforge.net/).

The structure of hUGDH in complex with NAD^+^ fragments and UDP-glucose was solved by molecular replacement and refined against 2.8 Å resolution data (Table S1) [32]. The crystal asymmetric unit contains two open hexamers arranged as a closed dodecamer (Fig. 2). We refer to the twelve hUGDH subunits as subunits A to F and G to L, representing the two open hexamers of the dodecamer within the crystal asymmetric unit, respectively. Twelve-fold non-crystallographic restraints were maintained up to the last cycle of refinement. The placement of residues, discussed below, stand up to twelve-fold averaging of the electron density maps providing a higher level of confidence in these features than would normally be afforded to a structure refined against 2.8 Å resolution data.

### Three-domain protomer

**Figure 3:**
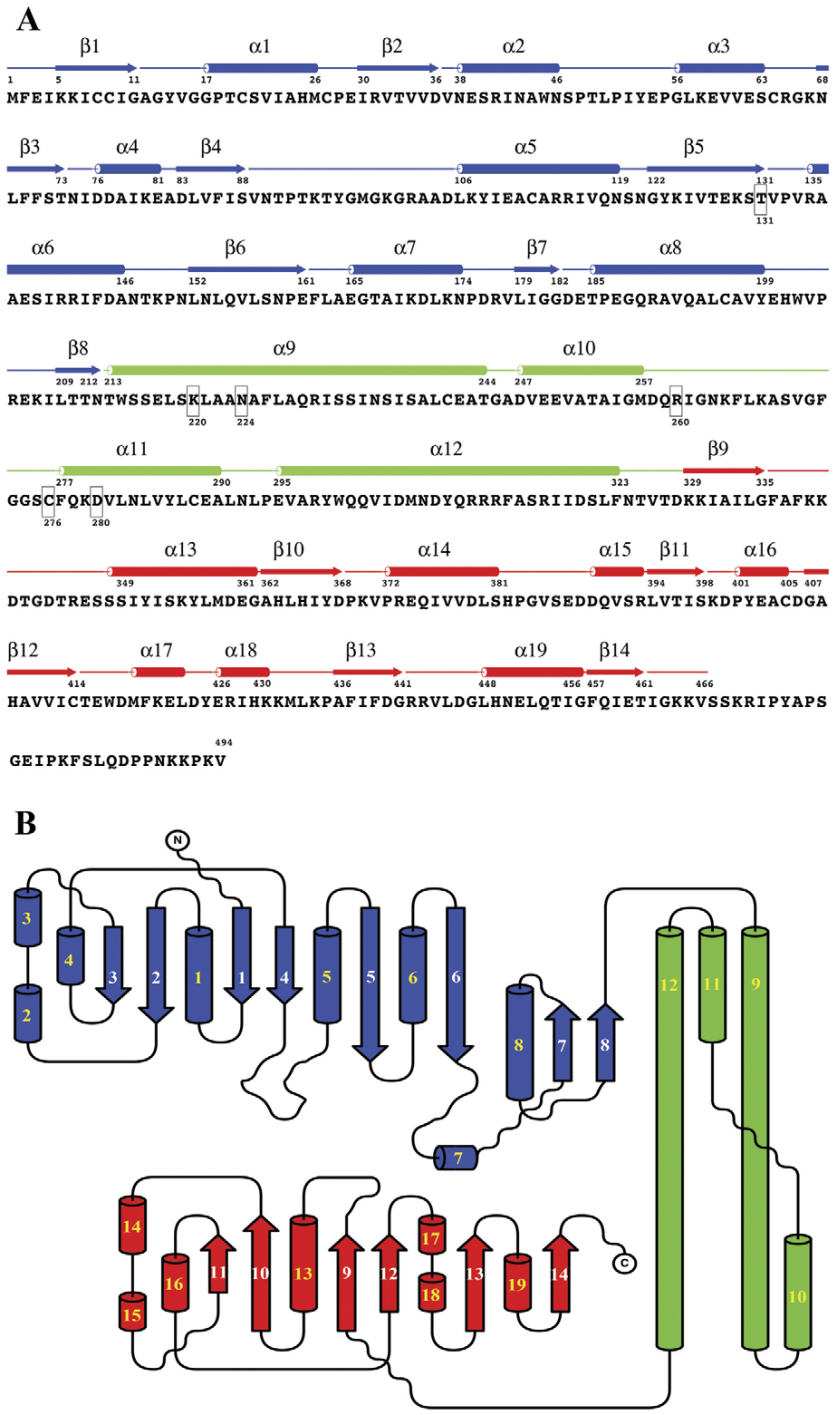
Secondary structure and topology of the hUGDH protomer. (A) Secondary structure elements shown above the hUGDH amino acid sequence in domain colors (N-terminal, blue; central, green; C-terminal, red). Important active site residues are boxed. (B) Topology of the protomer. The figures were generated in ALSCRIPT [49] and TOPDRAW [50].

The hUGDH protomer consists of three domains. The N- and C-terminal domains are structurally homologous α/β structures, comprising core β-sheets sandwiched between α-helices (Fig. 2A, blue and red, respectively). These two domains sit upon an extended α-helical central domain (Fig. 2A, green). The topologies of the α/β domains are characteristic of the dinucleotide-binding Rossmann fold (Fig. 3) [33]. The six-stranded parallel β-sheet (β1–β6, residues 1–161) of the N-terminal domain is followed by an additional β-α-β unit (β7, α8 and β8, residues 179–212). The β-strands of this unit are antiparallel with respect to the Rossmann fold (Fig. 3B). The NAD^+^ fragments (Fig. 2A, yellow) are bound in the cleft between strands β2 and β4 and lying across the β1-α1 loop. This would position the nicotinamide portion, which is not visible in this structure, at the substrate-binding site formed at the interface between the three domains. The α/β C-terminal domain (residues 329–466) consists of a six-stranded sheet (β19–β14) packed with α-helices on both sides (Fig. 3B). This second Rossmann fold is responsible for binding the UDP moiety of UDP-glucose (Fig. 2A, purple), positioning the sugar moiety at the active site. The central domain is comprised of four helices (α9–α12, residues 213–323). The first and last of these helices are extended and cross to form the scaffolding that positions the N- and C-terminal domains, respectively.

### Tightly associated dimeric unit

**Figure 4:**
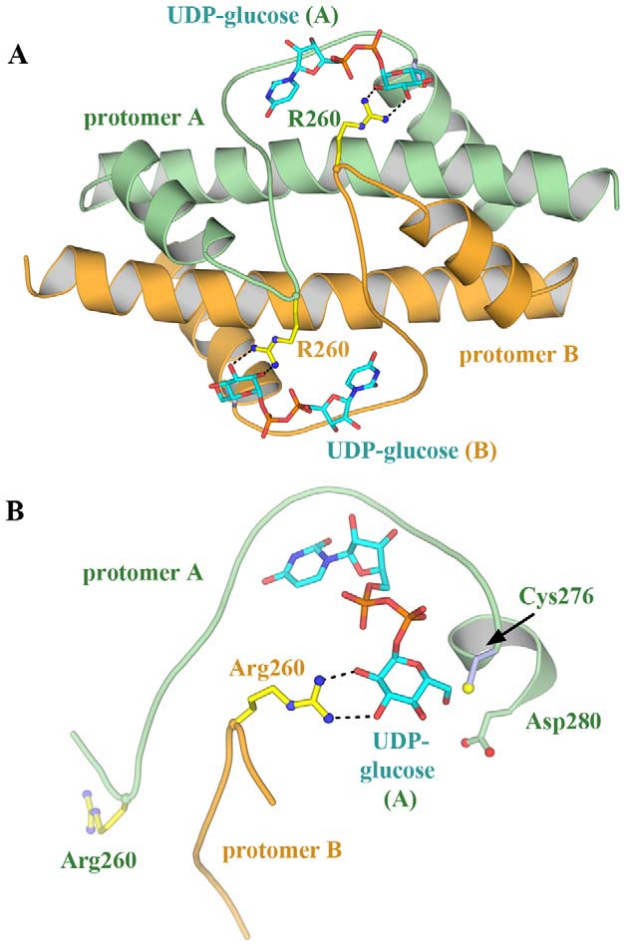
Cooperativity within the hUGDH dimeric unit. (A) Residues 265–280 wrap around and orient UDP-glucose in one subunit while Arg260 forms two hydrogen bonds to the glucose moiety of UDP-glucose in the second protomer. Hence, residues 260–280, which include active site residues Cys276 and Asp280, may act as a sensor by transmitting the state of one UDP-glucose binding site to the other protomer at the dimer interface. (B) Close up of the UDP-glucose binding site.

The protomers are arranged in pairs associated through dimerization of the central domains in an interface of 2600 Å^2^ (Fig. 2B). Arg260 situated in a loop within the central domain of one protomer reaches across to contact and position the glucose from the UDP-glucose within the second protomer (Fig. 4). The subsequent polypeptide chain, residues 265–280 that includes active site residues Cys276 and Asp280 (Fig. 1), wraps around the UDP-glucose from the first protomer forming multiple contacts. Hence, the occupancy of the UDP-glucose binding site within one half of the dimeric unit will be sensitive to that in the second half, suggesting cooperativity within the dimeric unit [28]. Furthermore, the dimerization interface may provide a platform to communicate more global conformational changes such as flexing of the central domains. The overall fold of the hUGDH protomer and the dimer interaction closely resemble that of the obligate dimer of UGDH from S. pyogenes [27].

### Open hexamer

Three copies of the hUGDH dimeric unit are arranged as a hexamer, through interactions between helix and loop structures in the N-terminal and C-terminal domains on neighboring protomers (Fig. 2C). The extended loop and α5-helix between β4–β5 (Fig. 3) and α6-helix from the N-terminal domain interact with the α13–β10 loop and α17–β13 loop that includes the α18-helix from C-terminal domain of the next protomer, respectively, while both N-terminal regions interact with the linker α12–β9 between the central and C-terminal domains. The interface area is approximately 700 Å^2^ between any two protomers in these inter-dimer contacts. An interaction from Asn292 to Asn292 links the central domains between pairs of dimers (A–F, B–C, G–L and H–I). The amino acid sequences at the inter-dimer contact regions show significant variability in comparison to the S. pyogenes obligate dimer, displaying three insertions, one deletion and different mainchain structures. The present structure has opened up to form an asymmetric hexamer (open hexamer) in comparison to the symmetric hexamer [28], PDB code 2Q3E (closed hexamer) (Fig. 2D). Despite these large differences in quaternary structure, the protomer structures from the open and closed hexamers show only small variations in conformation (RMSD of 0.83 Å, Table S2A) and similar interdomain arrangements (Table S2E). This compares with an RMSD of 0.18–0.43 Å for protomers within the open hUGDH hexamer. Superimposition of the individual domains of closed and open hexamer protomers does not reveal a significant conformational difference in C-terminal domains (RMSD = 0.41 Å, Table S2D) or the central domains (RMSD = 0.35 Å, Table S2C), whereas a comparison between N-terminal domains showed a slightly larger deviation (RMSD = 0.93 Å, Table S2B). These data indicate that the conformational difference detected from the whole hUGDH mainly results from structural plasticity in the N-terminal, NAD^+^-binding domain. Comparison of the open and closed hexamer protomers to reaction intermediate-bound and apo forms of the hUGDH shows larger variation (Tables S2A–E). In these structures, the separation of the N- and C-terminal domains slightly varies through rotation on the central domain backbone. However, the individual domains follow a similar pattern of relatedness to the open hexamer protomer as was determined for the closed hexamer. In contrast, the open hexamer structure determined here adopts the conformation observed for UDP-α-D-xylose bound UGDH [31] (RMSD = 0.29–0.34 Å between the protomers). Hence, this open hexamer UGDH conformation can interact with both the substrate and inhibitor.

### Asymmetric unit dodecamer

The asymmetric unit, within the crystal, contains two open hexamers arranged in a slightly twisted manner so that the open edges of the two hexamers (Fig. 2C). The areas of interaction surfaces are 220 Å^2^ (C∶K and E∶I) and 280 Å^2^ (D∶L and F∶J). Such contact areas are in the range observed for crystal contacts rather than for protein-protein interactions. Size exclusion analysis of hUGDH by FPLC, dynamic light scattering, and electron micrographs are consistent with the hexameric structure [34], [35], [36]. However, sedimentation velocity experiments suggest that apo-UGDH is a complex mixture of dimers, hexamers and small amounts of monomers and tetramers [31]. Furthermore, a single mutation (Lys339Ala) or double mutation (Ala222Gln and Ser233Gly) in hUGDH converts the hexamer to a dimer [34], [37]. These residues do not lie at the inter-dimer interfaces. Taken together, these data indicate that the hexameric structure is not highly stable. Hence, we suggest that the open hexamer conformation observed here results from the solution conditions and state of the NAD^+^ and is unlikely to be a major substrate-bound conformation under physiological conditions, nonetheless it provides valuable insight into the enzyme function.

### NAD^+^ binding

**Figure 5:**
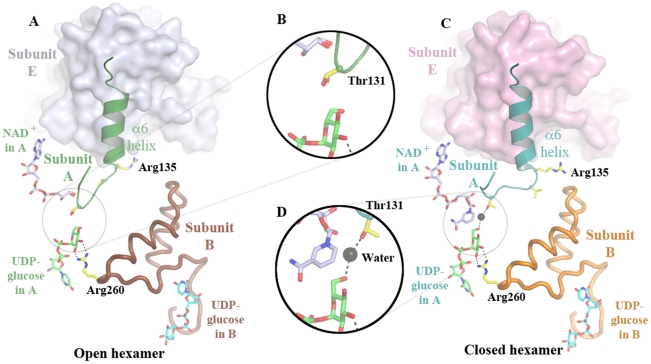
Cooperativity within the hUGDH trimeric unit. Here, movement of the protomer A Thr131 loop into the active water-binding site is associated with an adjustment to the α6-helix, which mediates contact to the next protomer E in the trimeric unit. (A) Portions of protomers A (green), B (brown) and E (gray surface) from the open hexamer. (B) Close up of Thr131 in the active water-binding site. (C) Portions of protomers A (teal), B (orange) and E (pink surface) from the closed hexamer. (D) Close up of the active water-binding site. Movie S3 shows a morph between these two structures.

Detailed comparison of the NAD^+^ conformations in this structure and those previously reported for the closed hexamer (2Q3E [28]) indicates striking differences. In the closed hexamer, the structure of each NAD^+^ molecule bound to every hUGDH protomer is well defined. The NAD^+^ molecules in the present open hexamer structure are partially disordered (Fig. 5A and Figure S1A). Similarly, disordered nicotinamide rings were found in the inhibitor-bound and thiohemiacetal-bound structures [28], [31]. The partially disordered NAD^+^ has direct consequences for protein structure around the NAD^+^-binding site. In comparison to the closed hexamer, the open hexamer residues Ser130 and Thr131 have made a large switch toward the interior of the NAD^+^-binding site to occupy the space vacated by the disordered nicotinamide moiety and the absent catalytic water (Fig. 5). These conformational differences with respect to the closed hexamer are observed in all twelve protomers and are not dependent on position within the open hexamer. This indicates that the conformational changes are likely to be dependent on the occupation of the NAD^+^ and catalytic water binding sites rather than induced by the opening of the hexamer. The Thr131 loop movement at the NAD^+^-binding site results in long-range conformational changes of the loop-α6 helix unit between β5–β6 (residues 129–152) in the N-terminal domains of each protomer within the dodecamer (Fig. 5). This region includes an intersubunit contact site in the trimer unit (Fig. 2C). We speculate that, under the crystallization conditions, NAD^+^-binding site induced rearrangement of the β5-α6-β6 region placed strain on the hexamer causing it to open.

### UDP-glucose binding

**Figure 6:**
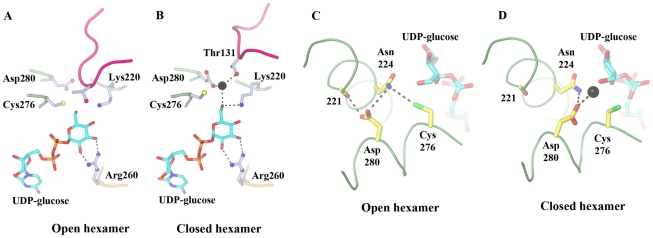
The UDP-glucose binding site. (A, B) The active site conformations of the open and closed hexamers, respectively. (C, D) The position and hydrogen-bonding pattern of Asp280 in the open and closed hexamers, respectively. The active water molecule is shown as a black sphere. Movie S4 shows a morph between these two structures.

The UDP-glucose is well defined in the active site (Fig. 6) and the UDP portion is essentially identical in all 12 crystallographic protomers. However, the oxygen of the C6′ hydroxyl shows poor density, and hence, its positioning was determined by the general fitting of the glucose ring to the electron density (Figure S1B). The glucose moiety appears to display a distribution of positions ranging from one in which the C6′ hydroxyl is within bonding distance to Cys276 (as in subunit A, Fig. 6A) to an orientation where the C6′ hydroxyl is close to Lys220 (subunit L, Figure S1C). Movement of residues Ser130 and Thr131 into the space occupied by the missing NAD^+^ nicotinamide ring has important consequences for the active water molecule and active site residues. Firstly, Asp280 coordinates the catalytic water in the closed hexamer protomers (Figs. 1 and 6b). In the open hexamer protomers there is no bound active water. Instead, Thr131 occupies the former location of the active water and causes Asp280 to turn away from the UDP-glucose (Fig. 6C,D and Figure S2). Asp280 (atom OD2) lies within hydrogen-bonding distance (mean distance 2.72 Å) of the mainchain carbonyl oxygen of Leu221, indicating that the acid is protonated. The sulphur atom of the catalytic Cys276 is also significantly displaced, relative to the open hexamer protomers, and turns towards Asn224 (mean SG to ND2 distance 3.51 Å), suggesting that Cys276 is not protonated.

## Discussion

Enzymes, particularly in higher organisms, display a tendency to form large multimers. Cooperativity provides a possible explanation for such refinement during evolution. Through linking reaction cycles between subunits, and hence sharing energy, an enzyme has the opportunity to alter energy barriers at points in the reaction cycle that would not be available to an isolated protomer. The present structure of hUGDH displays an asymmetric open hexameric conformation. This open conformation was previously observed for the non-productive inhibitor-bound hUGDH. Here, we speculate that this distorted substrate-bound conformation is unlikely to be that of a native conformation but rather one that has been pushed to extreme through solution conditions and ligand binding. Nevertheless, conformational differences between the open form and the closed forms of hUGDH provide important insights into the structural basis of cooperativity within the hexamer.

UGDH displays a high degree of cooperativity between subunits [1] operating as a “trimer of dimers” in which only three subunits are simultaneously active. A structural indication of this trimer of dimers is observed in the thiohemiacetal-trapped E161N hUGDH mutant structure (PDB 3KHU), which shows partial disorder of NAD^+^ in only one trimer [28]. Within the dimeric unit (Fig. 4), residues from both protomers are in contact with each UDP-glucose in the two active sites [28]. Indeed, Arg260 from one subunit binds to the glucose moiety in the second subunit while residues 265–280, which include active site residues Cys276 and Asp280, surround the UDP-glucose in the first subunit. Hence, the occupancy of one UDP-glucose binding site will have direct implications for the occupation and reaction state of the second subunit-binding site within the dimeric unit. We suggest that residues 260–280 act as a reaction sensor that splits the six protomers into the two sets of three.

Synchronization of the reaction within a trimeric unit is not achieved through a direct mechanism, since the protomers do not contact the UDP-glucose in their trimerically related neighbors. However, the open hexamer structure suggests a possible communication route. In the open hexamer protomers, Thr131 and the associated loop has moved into the unoccupied active water and nicotinamide ring of NAD^+^ binding sites, resulting in a shift of the α6 helix region (Fig. 5). This N-terminal domain motif contains an intra-trimer subunit contact site for the C-terminal domain of the next protomer. Hence, the reaction state at one active site maybe communicated in a clockwise direction to the adjacent protomer (Fig. 2D). We speculate that this may cause the intra-subunit flexing of the C-terminal domain relative to the N-terminal domain [28] within the adjacent protomer, relaying the information to the substrate-binding site, which lies at the junction of the three domains (Fig. 2). Such a mechanism provides an explanation as to why the enzyme operates as a trimer of dimers. Sequentially, one trimer may provide the scaffolding on which the other trimer can perform reaction-specific conformational changes without risk of hexamer disintegration. Hence, we propose that Thr131 and associated loop acts as a second catalytic sensor, in this case synchronizing the radially associated subunits.

The residue arrangement at the active site of the open hexamer is broadly consistent with the catalytic mechanism (Fig. 1), positioning of the C6′ hydroxyl of the UDP-glucose near to Lys220 and Cys276. However, Asp280, which coordinates the active water, has turned away from the substrate into a non-productive conformation where it contacts the mainchain carbonyl oxygen of Leu221 (Fig. 6C,D and Figure S2). Asp280 is required to be protonated to adopt this conformation. Furthermore, Thr131 has moved into the active water site obscuring access to UDP-glucose from the NAD^+^ direction (Figs. 5a and 6a). Covalent attachment of reaction intermediates has been shown to not be necessary to lock reaction intermediates in the substrate-binding site during the exchange of NADH for NAD^+^ [38]. As such, we propose that the movement of the Thr131 loop coupled with domain-domain movements protect substrate intermediates during NADH dissociation.

In the reaction cycle, UGDH initially binds to UDP-glucose and then sequentially binds, reduces, and releases two NAD^+^ molecules before releasing the product, UDP-glucuronic acid (Fig. 1). Mutation of Thr118 to Ala in hUGDH led to a 8-fold reduction in kcat, demonstrating non-essential mechanistic importance of this threonine [28]. The conformation of Thr131 at the active site in the dodecamer may have relevance at two points in the reaction cycle. Firstly, after the reduction of the first NAD^+^, Thr131 may move to protect the intermediate during NADH∶NAD^+^ exchange and allow Asp280 to reorient in order to lose a proton and subsequently repolarize the active water. This movement and repolarization would be applicable to either of the proposed mechanisms (Fig. 1, Ia and Ib). Secondly, the structure of hUGDH bound to UDP-glucuronic acid (PDB code 2QG4 [28]) contains the ordered active water molecule bound to Asp280. This water molecule is a prime candidate to become integrated into UDP-glucuronic acid during the reaction (Figure S1D), and as such, this structure may be a mimetic of the substrate-bound form rather than the product-bound form. In contrast, the open hexamer structure suggests that during the reduction of the second NAD^+^, Thr131 may track this active water as it incorporates into UDP-glucuronic acid, and in turn drive NADH dissociation (Fig. 1, III and IV). Interestingly, Egger and coworkers were not able to crystallize apo-hUGDH, however, yet were successful in crystallizing the T131A mutant in the apo form, reinforcing the link between Thr131 and ligand binding [28]. Hence, we propose that the present structure of hUGDH represents a trapped intermediate conformation that provides valuable insight into the reaction mechanism. The observed conformational plasticity indicates that the inhibitor UDP-α-D-xylose functions by trapping the hUGDH protomers in an intermediate reaction state leading to the stalling of the enzyme and placing strain on the symmetrical hexameric structure. Further stabilization of this conformation will provide an attractive strategy for inhibitor drug discovery.

## Materials and Methods

### Protein production

Recombinant wild-type hUGDH was purified and the activity verified spectrophotometrically by measuring the reduction of NAD^+^ in the presence of UDP-glucose [39]. A deletion mutant was generated that lacked amino acids 488–494, which was also purified via this protocol [32].

### Crystallization and Data Collection

Crystals obtained from the full-length protein only diffracted to 6 Å. Truncating the protein by seven residues produced diffraction quality crystals. These crystals were grown by equilibrating the protein solution (10 mg ml−1 in 50 mM Tris-HCl, pH 8.0, 0.5 mM EDTA, 10 mM mercaptoethanol, 5 mM UDP-glucose, 2 mM NAD^+^, and 7% (v/v) glycerol) mixed 1∶l with the reservoir solution (0.2 M ammonium sulfate, 0.1 M Na cacodylate, pH 6.5, and 21% PEG 8000) and incubated over 0.5 ml of reservoir solution at 295 K. The crystals grew within two days [32]. Mass spectrometry analysis confirmed that the product (UDP-glucuronic acid) could be detected on incubation of UDP-glucose with hUGDH under the crystallization conditions in the absence of PEG 8000 and ammonium sulphate, confirming that the enzyme was active at the pH of the crystallization condition. Diffraction data to 2.7 Å resolution were collected at the 4A Macromolecular Crystallography Wiggler Beamline of the Pohang Accelerator Laboratory (Pohang, Korea) using the X-ray beam at a single wavelength (1.1272 Å) and 1° oscillations. The data set was indexed and processed with the CCP4 suite of programs [40]. Data processing statistics and crystal characteristics are reported in Table S1.

### Molecular Replacement and Refinement

Initial attempts to solve the structure by molecular replacement, using the bacterial structure (PDB code 1DLI) as the search model, failed [27], [32]. Using this model, the solution with the highest correlation coefficient suggested a tetrameric arrangement. The structure was subsequently solved using molecular replacement on the release of the hUGDH hexamer structure (PDB code 2Q3E [28]) with the program PHASER [41]. The dimeric unit was used as the search model. The solution, which includes six copies of the search model, was unambiguous with Z-Score values for rotational and translational functions of 20.8 and 144.4, respectively. The Patterson function reveals a significant non-origin peak that is 50.2% of the origin peak, consistent with a pseudo translational symmetry for the two open hexamers. A Matthews coefficient of 2.98 Å3 Da−1 can be derived for a dodecamer, this relates to 58.73% solvent content. Refinement was initially was carried out using an automated protocol with NCS restraints in PHENIX [42]. Careful model rebuilding subsequent rounds of refinement, in REFMAC and COOT [43], [44], in which the NCS restraints were released, resulted in the current model (Table S1). The final model includes ordered residues 1–466. This range matches that found to be ordered in the structures of full length hUGDH∶inhibitor complexes [31] and of the truncated (1–467) hUGDH complexes [28], which showed normal enzymatic activity. The coordinates and merged reflection data for hUGDH have been deposited in the RCSB Protein Data Bank with accession code 3TDK.

## Supporting Information

Figure S1 **hUGDH ligand binding.** 2Fo-Fc OMIT map electron density contoured at 1 σ for (A) the NAD^+^ fragments and (B) UDP-glucose. (C) The conformation of UDP-glucose within the active site of subunit L. (D) The conformation of UDP-glucuronic acid (PDB code 2QG4) in the active site. Note: This structure contains an ordered active water molecule that would normally become integrated into UDP-glucuronic acid during the reaction. Hence, the structure may mimic the substrate-bound form rather than the product-bound form of the enzyme.
(TIF)

Figure S2 **The conformation of active site residues.** 2Fo-Fc OMIT map electron density contoured at 1 σ around the active site residues Asn 224, Cys276 and Asp280.
(TIF)

Table S1 **Data collection and refinement statistics for dodecameric hUGDH.**
(DOC)

Table S2 **Comparison of hUGDH structures.** RMS deviation of Cα positions between hUGDH domains and protomers. (A) Protomers. (B) N-terminal domains. (C) central domains. (D) C-terminal domains. (E) Distances between residues in the N-terminal (Gly166 and Tyr53) and C-terminal (Asp341 and Gly343) domains. Protomers can adopt either an open or a closed conformation indicated by # and *, respectively. PDB code 2Q3E is a hexamer that represents the reaction start that contains UDP-glucose and NAD^+^ in the ligand binding sites. The 3KHU hexamer mimics the thiohemiacetal intermediate and contains UDP-glucose in the NAD^+^ binding site. The PDB code 2QG4 hexamer represents the product bound structure with UDP-glucuronic acid bound at UDP-glucose binding site. This structure contains NAD^+^ in one trimer (subunits B, D, F, H) and a cleaved version of NAD^+^ without the nicotinamide ring in the second trimer (subunits A, C, E, G) of the dimer-of-trimers that comprise the hUGDH hexamer, providing structural confirmation of the three site biological activity. The PDB code 3ITK hexamer mimics the unbound state of hUGDH in which both ligand-binding sites do not contain the appropriate ligand. The 3ITK protomers are in an open conformation with the exception of subunit F, which is in the closed conformation. F participates in a dimer interaction with E, the most open of the 3ITK protomers, suggesting structural cooperativity within the dimeric unit.
(DOC)

Movie S1 **Morph between the docecamer and two copies of the closed hexamer (2Q3E).** This morph is only intended to give an impression of the scale of conformational change needed to move between these two structures.
(MOV)

Movie S2 **Morph between the open hexamer and the closed hexamer (2Q3E).** The pink helix highlights the α6-helix and the preceding Thr131-containing loop. This morph is only intended to give an impression of the scale of conformational change needed to move between these two structures.
(MOV)

Movie S3 **Morph between protomers of the open and the closed (2Q3E) hUGDH hexamers focused at the active site.** The movie shows the flipping of Asp280 (yellow), movement of Thr131 (pale green) into the active water-binding site (grey sphere), flipping of Trp214 (lime) and Arg135 (pale green). This morph is only intended to give an impression of the scale of conformational change needed to move between these two structures.
(MOV)

Movie S4 **Morph between protomers of the open and the closed (2Q3E) hUGDH protomers within a trimeric unit.** Here, movement of the Thr131 loop into the active water-binding site is associated with an adjustment to the α6-helix (all in yellow), which mediates contact to the next protomer in the trimeric unit. N-terminal, central and C-terminal domains are shown in blue, green and red, respectively. This morph is only intended to give an impression of the scale of conformational change needed to move between these two structures.
(MOV)
