# Supplementary figures and images for: Structural Basis of Cooperativity in Human UDP-Glucose Dehydrogenase
Source: PLoS One. 2011 Oct 3;6(10):e25226. doi: 10.1371/journal.pone.0025226 (PMC3184952; doi:10.1371/journal.pone.0025226)

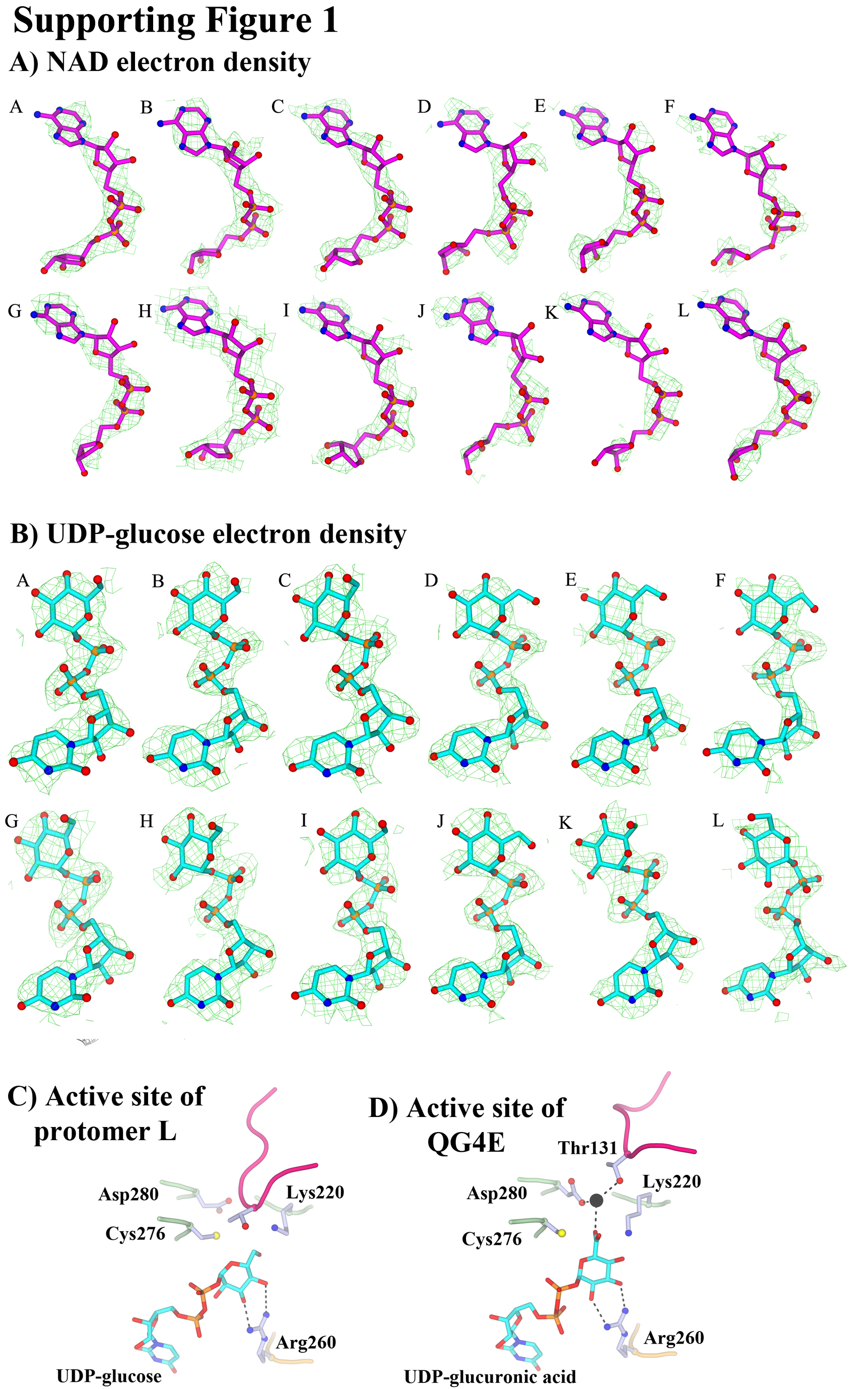

Supplement: Figure S1 — hUGDH ligand binding. 2Fo-Fc OMIT map electron density contoured at 1 σ for (A) the NAD+ fragments and (B) UDP-glucose. (C) The conformation of UDP-glucose within the active site of subunit L. (D) The conformation of UDP-glucuronic acid (PDB code 2QG4) in the active site. Note: This structure contains an ordered active water molecule that would normally become integrated into UDP-glucuronic acid during the reaction. Hence, the structure may mimic the substrate-bound form rather than the product-bound form of the enzyme. (TIF) [file pone.0025226.s001.tif]

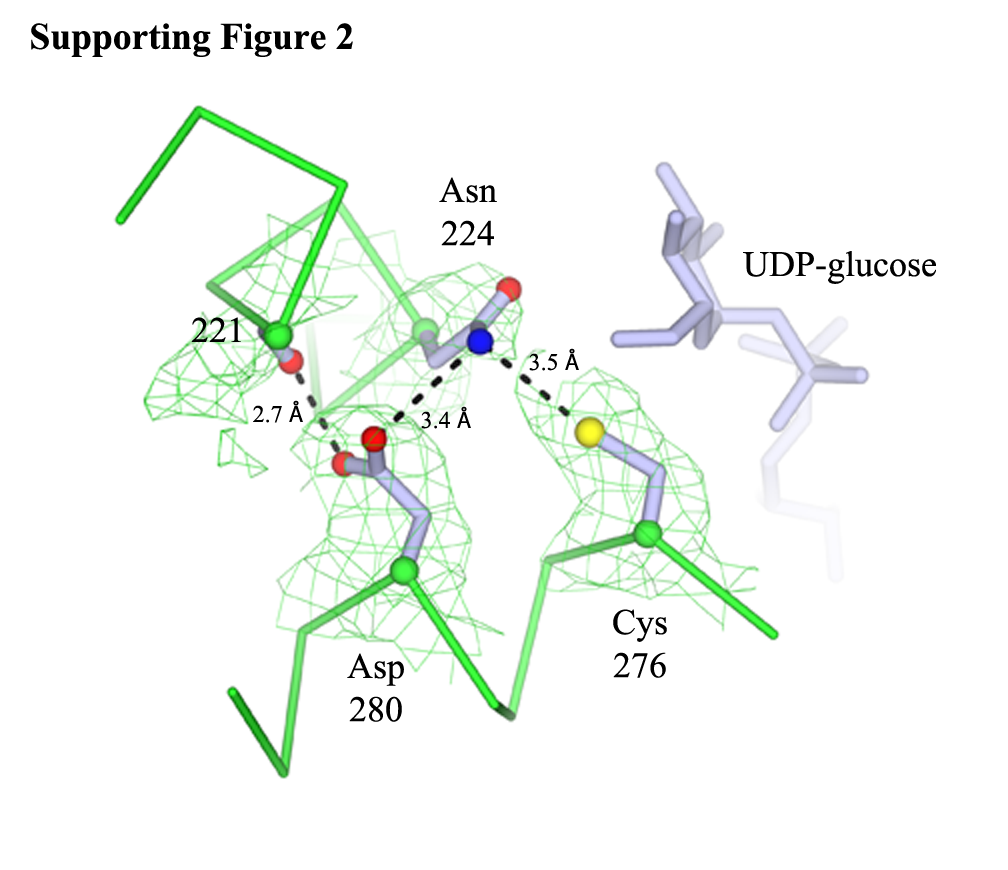

Supplement: Figure S2 — The conformation of active site residues. 2Fo-Fc OMIT map electron density contoured at 1 σ around the active site residues Asn 224, Cys276 and Asp280. (TIF) [file pone.0025226.s002.tif]
