# Supplementary material for: Structural Basis of Cooperativity in Human UDP-Glucose Dehydrogenase
Source: PLoS One. 2011 Oct 3;6(10):e25226. doi: 10.1371/journal.pone.0025226 (PMC3184952; doi:10.1371/journal.pone.0025226)
Supplement: Table S1 — Data collection and refinement statistics for dodecameric hUGDH. (DOC) [file pone.0025226.s003.doc]

| **Data-collection statistics**  **(Values in parentheses are for the highest resolution shell)** |  |
| --- | --- |
| Wavelength (Å) | 1.1272 |
| Resolution range (Å) | 20-2.8 (2.95-2.8) |
| Space group | P2_1_2_1_2_1_ |
| Cell parameters (Å) | 173.132  191.177  225.807 |
| No. of observed reflections | 871251 |
| No. of unique reflections | 176566 |
| *R*merge(*I*) (%) | 12.7 (51.7) |
| Completeness (%) | 96.1 (90.5) |
| Redundancy | 4.9 (3.7) |
| *I*/sig(*I*) | 8.0 (2.5) |
| Refinement statistics |  |
| Resolution range (Å) | 20.0-2.8 |
| No. of reflections used | 167388 |
| Size of *R*free set (%) | 5 |
| *R*cryst/*R*free (%) | 23.0 (34.0)/26.1 (39.0) |
| R.M.S.D. |  |
| Bonds (Å) | 0.014 |
| Angles (°) | 1.46 |
| Ramachandran plot |  |
| Residues in most favored regions (%) | 96.7 |
| Residues in additional allowed regions (%) | 3.3 |
| Residues in outlier region (%) | 0 |
| Estimated coordinate error (Å) |  |
| Coordinate error (Maximum-Likelihood based) | 0.304 |
| Coordinate error (DPI) | 0.373 |
| Mean *B* factor protein, UDP-glucose, NAD^+^ (Å^2^) | 33.8, 42.7, 54.0 |
