# Supplementary material for: Structural Basis of Cooperativity in Human UDP-Glucose Dehydrogenase
Source: PLoS One. 2011 Oct 3;6(10):e25226. doi: 10.1371/journal.pone.0025226 (PMC3184952; doi:10.1371/journal.pone.0025226)
Supplement: Table S2 — Comparison of hUGDH structures. RMS deviation of Cα positions between hUGDH domains and protomers. (A) Protomers. (B) N-terminal domains. (C) central domains. (D) C-terminal domains. (E) Distances between residues in the N-terminal (Gly166 and Tyr53) and C-terminal (Asp341 and Gly343) domains. Protomers can adopt either an open or a closed conformation indicated by # and *, respectively. PDB code 2Q3E is a hexamer that represents the reaction start that contains UDP-glucose and NAD+ in the ligand binding sites. The 3KHU hexamer mimics the thiohemiacetal intermediate and contains UDP-glucose in the NAD+ binding site. The PDB code 2QG4 hexamer represents the product bound structure with UDP-glucuronic acid bound at UDP-glucose binding site. This structure contains NAD+ in one trimer (subunits B, D, F, H) and a cleaved version of NAD+ without the nicotinamide ring in the second trimer (subunits A, C, E, G) of the dimer-of-trimers that comprise the hUGDH hexamer, providing structural confirmation of the three site biological activity. The PDB code 3ITK hexamer mimics the unbound state of hUGDH in which both ligand-binding sites do not contain the appropriate ligand. The 3ITK protomers are in an open conformation with the exception of subunit F, which is in the closed conformation. F participates in a dimer interaction with E, the most open of the 3ITK protomers, suggesting structural cooperativity within the dimeric unit. (DOC) [file pone.0025226.s004.doc]

**Supporting Tables 2A-E**

| **Supporting Table 2A** Comparison of whole protomers (1-466) RMSD (Å) | | | | | | | |
| --- | --- | --- | --- | --- | --- | --- | --- |
|  | **PDB** | **Dodecamer** | **3ITK** | **3ITK** | **2QG4** | **2QG4** | **3KHU** |
| **Reaction state** | **(Subunit)** | **(A)*** | **(F)*** | **(A)#** | **(B)*** | **(A)#** | **(A)#** |
| **UDP-glucose** | **2Q3E (A)*** | 0.83 | 1.00 | 2.17 | 0.23 | 1.88 | 1.54 |
| **Thioester** | **3KHU (A)#** | 1.75 | 2.01 | 0.77 | 1.48 | 0.60 |  |
| **UDP-glucuronic acid** | **2QG4 (A)#** | 2.12 | 2.39 | 0.82 | 1.81 |  |  |
| **UDP-glucuronic acid** | **2QG4 (B)*** | 0.87 | 1.06 | 2.12 |  |  |  |
| **Apo** | **3ITK (A)#** | 2.31 | 2.53 |  |  |  |  |
| **Apo** | **3ITK (F)*** | 0.71 |  |  |  |  |  |

| **Supporting Table 2B** Comparison of N-terminal domains (1-211) RMSD (Å) | | | | | | | |
| --- | --- | --- | --- | --- | --- | --- | --- |
|  | **PDB** | **Dodecamer** | **3ITK** | **3ITK** | **2QG4** | **2QG4** | **3KHU** |
| **Reaction state** | **(Subunit)** | **(A)*** | **(F)*** | **(A)#** | **(B)*** | **(A)#** | **(A)#** |
| **UDP-glucose** | **2Q3E (A)*** | 0.93 | 0.70 | 0.64 | 0.21 | 0.46 | 0.56 |
| **Thioester** | **3KHU (A)#** | 0.76 | 0.57 | 0.31 | 0.56 | 0.29 |  |
| **UDP-glucuronic acid** | **2QG4 (A)#** | 0.86 | 0.65 | 0.41 | 0.46 |  |  |
| **UDP-glucuronic acid** | **2QG4 (B)*** | 0.95 | 0.73 | 0.65 |  |  |  |
| **Apo** | **3ITK (A)#** | 0.83 | 0.61 |  |  |  |  |
| **Apo** | **3ITK (F)*** | 0.72 |  |  |  |  |  |

| **Supporting Table 2C** Comparison of central domains (212-324) RMSD (Å) | | | | | | | |
| --- | --- | --- | --- | --- | --- | --- | --- |
|  | **PDB** | **Dodecamer** | **3ITK** | **3ITK** | **2QG4** | **2QG4** | **3KHU** |
| **Reaction state** | **(Subunit)** | **(A)*** | **(F)*** | **(A)#** | **(B)*** | **(A)#** | **(A)#** |
| **UDP-glucose** | **2Q3E (A)*** | 0.35 | 0.43 | 0.55 | 0.24 | 0.46 | 0.47 |
| **Thioester** | **3KHU (A)#** | 0.56 | 0.56 | 0.24 | 0.41 | 0.28 |  |
| **UDP-glucuronic acid** | **2QG4 (A)#** | 0.65 | 0.68 | 0.32 | 0.39 |  |  |
| **UDP-glucuronic acid** | **2QG4 (B)*** | 0.45 | 0.49 | 0.49 |  |  |  |
| **Apo** | **3ITK (A)#** | 0.62 | 0.60 |  |  |  |  |
| **Apo** | **3ITK (F)*** | 0.34 |  |  |  |  |  |

| **Supporting Table 2D**  Comparison of C-terminal domains (325-466) RMSD (Å) | | | | | | | |
| --- | --- | --- | --- | --- | --- | --- | --- |
|  | **PDB** | **Dodecamer** | **3ITK** | **3ITK** | **2QG4** | **2QG4** | **3KHU** |
| **Reaction state** | **(Subunit)** | **(A)*** | **(F)*** | **(A)#** | **(B)*** | **(A)#** | **(A)#** |
| **UDP-glucose** | **2Q3E (A)*** | 0.41 | 0.38 | 0.33 | 0.13 | 0.56 | 0.25 |
| **Thioester** | **3KHU (A)#** | 0.35 | 0.30 | 0.21 | 0.26 | 0.53 |  |
| **UDP-glucuronic acid** | **2QG4 (A)#** | 0.57 | 0.60 | 0.55 | 0.56 |  |  |
| **UDP-glucuronic acid** | **2QG4 (B)*** | 0.41 | 0.37 | 0.32 |  |  |  |
| **Apo** | **3ITK (A)#** | 0.37 | 0.28 |  |  |  |  |
| **Apo** | **3ITK (F)*** | 0.40 |  |  |  |  |  |

| **Supporting Table 2E** Subunit openness (Cα-Cα distances, Å) | | | | |
| --- | --- | --- | --- | --- |
| **Reaction state** | **PDB** | **Subunits** | **G166-D341, Å** | **Y53-G343, Å** |
| **Closed conformation *** |  |  |  |  |
| Dodecamer |  | A-L | 4.6-4.9 | 5.4-5.9 |
| Reaction start | 2Q3E | A-F | 4.6-4.7 | 5.5-5.7 |
| Product-bound mimetic | 2QG4 | B,D,F,H | 4.5-4.6 | 5.6-5.7 |
| Unbound | 3ITK | F | 4.5 | 6.0 |
| **Open conformation #** |  |  |  |  |
| Thiohemiacetal mimetic | 3KHU | A-F | 7.3-7.4 | 9.5-9.6 |
| Product-bound mimetic | 2QG4 | A,C,E,G | 7.6-7.7 | 10.1-10.6 |
| Unbound | 3ITK | A-E | 8.2-9.4 | 9.4-12.1 |
